# Homocysteine May Involve in the Pathogenesis of Behcet's Disease by Inducing Inflammation

**DOI:** 10.1155/2008/407972

**Published:** 2009-02-01

**Authors:** Selda Pelin Kartal Durmazlar, Ahmet Akgul, Fatma Eskioglu

**Affiliations:** ^1^Department of Dermatology, Diskapi Yildirim Beyazit Education and Research Hospital, Ministry of Health Ankara, 06110 Ankara, Turkey; ^2^Department of Cardiovascular Surgery, Turkiye Yuksek Ihtisas Education and Research Hospital, Ministry of Health Ankara, 06100 Ankara, Turkey

## Abstract

*Objective*. Our aim was to evaluate the significance of homocysteine (Hcy) in Behcet's disease (BD) and the association of elevated Hcy levels associated with the indices of inflammation in BD. *Methods*. Untreated 70 patients with BD and 33 healthy individuals were included into the study. Hcy, tumor necrosis alpha (TNF-*α*), C-reactive protein (CRP), and erythrocyte sedimentation rate (ESR) were evaluated with respect to activity and specific individual clinical manifestations of the disease. *Results*. Hcy levels were found significantly elevated in active BD when compared to inactive BD and healthy controls. Hcy levels were found to have high correlation with the number of active clinical manifestations increased. A significant positive correlation was found between serum Hcy and TNF-*α* levels, CRP, and ESR. Hcy was found to be the best predictor of TNF-*α* among other parameters. *Conclusion*. Hcy may involve in the pathogenesis of BD by inducing inflammation.

## 1. INTRODUCTION

In 1969, McCully first proposed homocysteine (Hcy) to be a cause of cardiovascular disease due to the observation that patients with homocystinuria, a metabolic disease which results in high Hcy levels, also displayed atherosclerotic plaques at autopsies [1]. High levels of Hcy were associated with endothelial damage and oxidative stress [2, 3]. Currently, there is a classification commonly used for the degree of hyperhomocysteinemia as follows: normal (5–14 *μ*mol/L), mild (15–30 *μ*mol/L), moderate (31–100 *μ*mol/L), and severe (>100 *μ*mol/L) [4]. As many epidemiologic studies supported, mild hyperhomocysteinemia was found risk factor for both venous and arterial thrombosis [5–8]. Hcy is an intermediary sulphydryl-containing amino acid formed during the conversion of methionine to cysteine. Its sulphydryl group can cause direct endothelial cytotoxicity, inhibition of glutathione peroxidase and nitric oxide, interference with clotting factor, and oxidation of LDL [9]. Additionally, high levels of Hcy cause lipid peroxidation, impaired vasomotor regulation, prothrombic surface, and, therefore, atherothrombogenesis [10].

Behcet's disease (BD), initially described by Behcet in 1937 [11], is a chronic relapsing vasculitis, involving both arteries and veins of various sizes. Thrombotic complications have been reported in approximately 10–40% of BD patients [12]. Endothelial dysfunction due to inflammation is considered to be an important factor of thrombosis in BD. Homocysteine is reported to enhance endothelial leukocyte interaction [13]. Some studies have shown that hyperhomocysteinemia might be assumed to be an independent and correctable risk factor for thrombosis in BD [14–19]. Moreover, the association between Hcy levels and endothelial dysfunction has been shown in patients with BD [20]. However, endothelial injury itself cannot clearly account the hypercoagulable status of BD because other vasculitis syndromes do not increase the risk of thrombosis [21]. Additionally, existence of several contradictory results with respect to serum Hcy levels leads the role of homocysteine in BD unclear [22]. As vitamin B6, vitamin B12, and folic acid can decrease homocysteine levels [23], treatment with these vitamins can be considered to reduce the risk of venous and arterial thrombosis. Therefore, it is important to find a clear picture of the role of Hcy, which is thought to induce proinflammatory cytokines [24] during the course of BD.

The present study was conducted to evaluate serum Hcy as a serological marker for the assessment of the activity of BD with respect to specific individual clinical manifestations. Serum levels of Hcy were compared with the values of C-reactive protein (CRP), an acute phase reactant, the erythrocyte sedimentation rate (ESR), and tumor necrosis alpha (TNF-*α*) which is an important proinflammatory cytokine. The results were controlled with the healthy individuals.

In patients with BD, the question of whether hyperhomocysteinemia depends on the inflammation has not been answered yet. Therefore, our aim was to evaluate whether elevated Hcy levels, an independent risk factor for both venous and arterial thrombosis, associated with proinflammatory process mediated by cytokines in patients with BD.

## 2. MATERIAL AND METHODS

### 2.1. Subjects

70 patients (40 males, 30 females; mean age of 33 ± 7.79) fulfilling the international study group criteria [25] for the diagnosis of BD and 33 healthy control subjects (17 males, 16 females; mean age of 30 ± 6.68) were included in this study. Patients with BD were selected from patients who were being followed up by the departments of dermatology and cardiovascular surgery, Behcet's Disease Unit or referred from other departments. Subjects were newly diagnosed or patients who were not taking any medicine regarding BD for some reason. The control subjects were age- and sex-matched healthy hospital staff. Complete blood count, serum glucose, urea, creatinine, total cholesterol, triglyceride, uric acid, alanine aminotransferase, aspartate aminotransferease, alkaline phosphatase, vitamin B12, and folic acid levels were evaluated in all of the subjects before being included into the study. Patients who had other type of illness such as autoimmune disease that could affect the cytokine levels or patients taking any medication affecting the immune system or patients with folic acid or vitamin B12 deficiencies, diabetes mellitus, hyperlipidemia, chronic hepatitis, renal failure, severe psoriasis, pernicious anemia, and chronic alcoholism which might affect Hcy levels were excluded from the study. Patients taking vitamin supplements in the previous 6 months were also excluded from the study. The study was approved by the local ethics institute. Informed consent was obtained from all subjects. Pathergy test was performed and the number of active manifestations recorded for each patient.

### 2.2. Determination of disease activity

Since accepted specific clinical activity scoring system and laboratory screening profile for BD has not been performed so far, the activity of patients was considered to have an active disease in case of the existence of two or more symptoms with worsening of clinical symptoms and lack of wellbeing at the time of the study. Behcet's disease activity index (BDAI) was also performed on our patients according to the method presented by Bhakta et al. [26] and described elaborately by Lawton et al. [27]. An overall disease activity scores derived from adding the scores according to the evaluation of the duration of each clinical features (from 0 to 4) and patients and clinicians impressions of the disease activity. As it was agreed that inclusion of erythrocyte sedimentation rate (ESR) and C-reactive protein (CRP) measurements would not add significantly to overall measurement of disease activity [27] and were not included as criteria in BDAI [26, 27], ESR and CRP were not used in the determination of the activation of the disease. The patients who had no symptoms regarding BD in four weeks period or less than two symptoms with a healing process and an overall wellbeing status were grouped in inactive BD.

### 2.3. Vascular examination

All patients and healthy volunteers were examined carefully for vascular involvement. Either venous or arterial system involvement was defined as present when confirmed by Doppler ultrasonography, radioisotope venography, magnetic resonance angiography, conventional angiography, or computerized tomography.

### 2.4. Serum homocysteine analysis

Serum homocysteine levels in the study groups were determined by fluorescence polarization immunoassay (FPIA) technique (Axis Biochemicals, ASA, Oslo, Norway). FPIA was run on an IMx analyzer (Abbott, Ill, USA). The 95% confidence interval of plasma Hcy level suggested by the manufacturer for healthy individuals is 4.45–12.42 *μ*mol/L and serum Hcy level is expected to be up to 10% higher than ethylenediamine tetraacetate (EDTA) plasma. Five cc blood samples were drawn using a 25 gauge needle from a peripheral vein, avoiding haemolysis in the morning hours after an overnight fasting and 30 minutes of supine rest and collected into 10 mL empty evacuated tubes without EDTA, heparin, or clot activators. Samples were centrifuged at 1000 × g for 10 minutes. The serum was separated in aliquots and immediately frozen and stored at −80°C within 60 minutes until use.

### 2.5. Detection of tumor necrosis alpha by enzyme-linked immunosorbant assay

The TNF-*α* level was measured with an enzyme-linked immunosorbant assay (ELISA) kit (Bender Medsystems, Vienna, Austria, Lot *#* 223/208-D1) with a detectable level set at 14 pg/mL. Five cc serum was separated by centrifugation at 1000 × g for 10 minutes and stored at −70°C until use.

### 2.6. C-reactive protein, erythrocyte sedimentation rate analyses

CRP was measured by a highly sensitive turbidimetric method using conventional biochemical automatic analyzer. The upper limit of the normal range was 5 mg/L. The ESR was determined by the classic Westergren method.

### 2.7. Statistical analysis

Results were analyzed using SPSS for Windows VER 11.5 (SPSS Inc., Chicago, Ill, USA). Each variant was evaluated by one sample Kolmogorov-Smirnov test for compatibility with normal distribution. As the data did not fit normal distribution, the nonparametric Kruskall-Wallis test for intergroups comparison and Mann-Whitney *U* test or Bonferroni corrected Mann-Whitney *U* tests were used for between-group comparisons as indicated. The relationship between variables was evaluated by Spearman correlation and regression analysis. Levels of significance set at 0.05 but in Bonferroni corrected test the significance threshold of 0.05 yielded to the value of 0.0167. Data are presented as median values and their individual ranges (in parenthesis).

## 3. RESULTS

Detected number of patients suffering from individual active clinical manifestations of the disease in various degrees was 31 with oral ulceration (44.28%), 23 with genital ulceration (32.85%), 18 with ocular involvement (25.71%), 37 with vascular involvement (52.85%), 25 with arthritis (35.71%), and 31 with cutaneous lesions like erythema nodosum (EN) and papulopustular eruption (PPE) (44.28%). Thirty five patients (50%) were found to have positive pathergy test. According to our evaluation scale, 34 patients were considered to have active disease with a median of 4 symptoms ranging between 2 and 5 and 36 patients to have inactive disease with a median of 1 symptom ranging between 1 and 2. Consistently, active group had a statistically significant high BDAI score with a median of 16 ranging between 9 and 25 compared to inactive group with a median of 2 ranging between 1 and 5 (*P* = .00) (Table 1). According to vascular examination, venous involvement (deep venous thrombosis with or without thromboflebitis) was found in 23 patients and either arterial occlusion or aneurysm was found in 14 patients in active group. 17 patients in inactive group had short duration of thromboflebitis with a wellbeing status.

In intergroup comparison of active BD, inactive BD, and healthy controls according to serum levels of Hcy, TNF-*α*, CRP, and ESR significant difference was detected for each parameter (*P* = .00 for each parameter) (Table 1). To determine which group/groups cause the difference, between-group comparisons performed. According to this, Hcy, TNF-*α*, CRP, and ESR levels were found elevated in active BD compared to inactive BD and healthy controls. Hcy, TNF-*α*, CRP, and ESR levels of inactive BD were found elevated compared to healthy controls. Hcy and TNF-*α* suggested having a strong association of disease activity (Table 1). Hcy and TNF-*α* were found to have high correlation with BDAI scores (*r* = 0.836, *P* = .00 and *r* = 0.924, *P* = .00, resp.) and the number of active clinical manifestations increased (*r* = 0.694, *P* = .00 and *r* = 0.842, *P* = .00, resp.) while ESR and CRP also showed a positive correlation with both parameters with relatively less significance (Table 2).

The evaluation of association between specific individual clinical manifestations and serum levels revealed increased Hcy levels in patients with oral ulcer (*P* = .03), genital ulcer (*P* = .005), the presence of positive pathergy test (*P* = .007), ocular lesion (*P* = .00), and vascular lesion of high significance (*P* = .00) (Table 3). No association was found between Hcy levels and EN/PPE and arthritis.

The serum levels of TNF-*α* were found to have strong association with oral ulcer (*P* = .00), genital ulcer (*P* = .00), the presence of positive pathergy test (*P* = .00), ocular lesion (*P* = .00), and vascular lesion (*P* = .000) (Table 4). No association was found between TNF-*α* levels and EN/PPE and arthritis.

The association of CRP with genital ulcer (*P* = .001), ocular lesion (*P* = .001), vascular lesion (*P* = .001), EN/PPE (*P* = .043), and the presence of positive pathergy test (*P* = .016) was found. No association was found between CRP levels and ocular lesion and arthritis (Table 5). ESR showed no significant association with any of the specific individual clinical manifestations (Table 6).

A significant positive correlation was found between serum Hcy and TNF-*α* levels (*r* = 0.89, *P* = .00), CRP (*r* = 0.645, *P* = .00) and ESR (*r* = 0.561, *P* = .00) (Table 2). Among parameters, Hcy was found to be the best predictor of TNF-*α* which was used as a dependent variable (Beta = 6.45, *R* ^2^ = 0.88, *P* = .00) (Table 7).

Serum levels of Hcy in active BD patients with vascular involvement were found significantly increased when compared in patients with active BD without vascular involvement. In addition, serum levels of Hcy in inactive BD with vascular involvement were found significantly increased compared to inactive BD (Table 8).

Serum levels of active BD patients with vascular involvement, oral ulcer, arthritis/arthralgia, EN/PPE and the presence of positive pathergy test were found significantly increased compared to inactive BD patients with the same clinical symptoms (Table 9).

## 4. DISCUSSION

The main pathology in BD is an inflammatory process of small arteries and veins and thrombosis as a result of vasculitis of the vaso vasorum [24]. Hyperhomocysteinemia has been shown to be a risk factor for thrombosis in BD in some recent studies [14–19]. In our study, serum levels of Hcy were found to have a positive correlation with BDAI scores and total number of active clinical manifestations. We also found significantly elevated levels of serum total Hcy in active BD when compared with inactive and control groups. Our results supported that total serum Hcy was a reliable marker for the activity of disease [17]. Aksu et al. [14] divided their patients with BD into two groups on the basis of thrombosis and they found hyperhomocysteinemia in patients with a history of thrombosis. However, Leiba et al. [22] could not find different serum total Hcy levels between patients with and without a history of thrombosis. The main limitations of this study were its retrospective nature and the long lag period between the thrombotic events and the time of analyses performed. Aksu et al. [14] evaluated their patients with respect to current clinical activity and 10 patients were accepted as clinically active, though they could not find association between serum Hcy levels and disease activity. The small sample size was a limitation of this study.

The main factor responsible for the increased frequency of thrombosis in BD is thought to be endothelial dysfunction caused by vascular inflammation [14, 15]. The association between Hcy levels and endothelial dysfunction and its correlation to the degree of endothelial damage has been shown in patients with BD [20]. Hcy generates superoxide and hydrogen peroxide, both of which have been linked to endothelial damage [15]. Hcy-induced vascular problems are thought to be multifactorial, including direct Hcy damage to the endothelium, enhanced lipid peroxidation and increased platelet aggregation by the effects on the coagulation system [15, 17].

In our study, the evaluation of association between specific individual clinical manifestations and serum levels revealed increased Hcy levels in patients with oral ulcer, genital ulcer, the presence of positive pathergy test and particularly with ocular and vascular lesions of high significance. No association was found between Hcy levels and EN/PPE and arthritis. Er et al. [15] showed the association of Hcy levels with disease activity and ocular BD. Other specific individual clinical manifestations were not assessed in this study. Ateş et al. [16] showed the association of Hcy levels with vascular involvement in BD. However, they could not find statistically significant difference between active and inactive diseases and association with mucocutaneous involvement in BD. On the other hand, Korkmaz et al. [28] reported no statistically significant association between vascular involvement and homocysteine levels, though 50% of patients with vascular involvement had hyperhomocysteinemia. Although the mechanism is unknown, all these findings indicate that Hcy associates with BD. However, the causes of these different study outcomes are not clear. The different sample sizes, different disease durations or study patients under treatment that might affect outcomes might have contributed. In addition, the question whether hyperhomocysteinemia depends on the inflammation or not remains to be unanswered.

Although the etiopathogenesis of BD has not yet been clarified, different immunological abnormalities have been reported and increased spontaneous secretion of TNF-*α*, Interleukin-6 (IL-6), and Interleukin-8 (IL-8) in monocyte cultures obtained from BD patients [29]. Necrotizing, neutrophilic (leukocytoclastic) obliterative perivasculitis, and venous thrombosis with lymphocytic and monocytic cellular infiltration of the veins, capillaries, and the arteries of all sizes and endothelial dysfunction are the hallmarks of BD [30]. Histopathological studies revealed cellular infiltration consisting of lymphocytes, plasmocytes, monocytes, and peripheral polymorphonuclear leukocytes (PMNs) in varying degrees, depending on the stage of lesion in BD [30, 31]. Since cytokines are involved in the regulation of functions of lymphocytes and phagocytes, they are playing important role in the pathogenesis of the disease [30, 31]. Although any organ system may be affected, there is high prevalence of venous or arterial thrombosis in BD and most clinical features are associated with systemic vasculitis. It has been suggested that vasculo-Behcet disease should be classified as a neutrophilic vasculitis targeting the vaso vasorum [32]. Aneurysm formation may be related to degeneration of arterial wall caused by inflammation of the vaso vasorum. As Hcy has profound effects concerning vascular lesions and thrombosis, vascular and ocular involvements in BD deserve special attention. Indeed, we found significantly elevated levels of Hcy with respect to the presence of vascular involvement in the intragroup comparisons of separately active and inactive BD patients. However, in our study Hcy was found to be able to evaluate the fluctuating activity of disease in an objective fashion and the number of additional organ systems affected. This finding may imply that Hcy cannot simply be assumed to be an independent risk factor for only vascular and ocular vaso-occlusive diseases in BD [14–19]. That is because most clinical features are thought to be associated with systemic vasculitis and dermal blood vessels embolized by thrombus are observed even at the sites of needle-prick reaction [30]. Furthermore, endothelial injury is a characteristic finding in BD and can be observed even in patients without any clinical vascular involvement [24, 33, 34]. These data provides insight as to why Hcy is able to detect overall disease activity with respect to different system involvements and why ascribed significance of Hcy is not limited only to the individual clinical manifestations of vascular or vaso-occlusive ocular involvements? Consistently, we found the association of Hcy not only with vascular and ocular vaso-occlusive disease but also with genital ulceration, positive pathergy test, and oral ulcers in BD patients, though with less significance. Therefore, our results not only support the suggestion that Hcy is a risk factor for vascular and ocular vaso-occlusive diseases in BD [15, 16] but also identify Hcy as a risk factor for the development of most clinical features that are known to be associated with vasculitis.

We found a significant positive correlation between serum Hcy, TNF-*α* and CRP levels, and ESR with relatively less significance. Hcy was found to be the best predictor of TNF-*α* among other parameters. In other words, Hcy was found to explain 88% of TNF-*α* change while CRP and ESR were found to explain 39% and 14% of TNF-*α* change, respectively. The significance of Hcy in BD represents either vascular endothelium damaged by Hcy or immune system stimulation by Hcy, our study may demonstrate the link between Hcy and inflammation pathways in BD. That is because TNF-*α*, CRP, and ESR are indices of inflammation. Although there are conflicting results in the literature with respect to the reliability of ESR and CRP as markers to detect the activity of BD and they are currently not involved as criteria in BDAI scores [27], CRP deserves special attention because it has a strong predictive power of vascular events [35]. We found both of them were elevated in the active stage of the disease. However, unlike CRP, ESR showed no significant association with any of the specific individual clinical manifestations. Relatively large sample size of our patients with vascular involvement might be a factor affecting our study outcome in favor of the significance of CRP in BD.

Chemotactic and phagocytic activities of neutrophils in patients with BD have been reported to be high [31] and TNF-*α* is a major factor modulating inflammatory responses which is known to be increased in inflammatory diseases. Recent studies showed increased TNF-*α* level in BD patients, especially in the exacerbation period [24, 30]. Synthesis of CRP in the liver is induced by several proinflammatory cytokines including TNF-*α*. This marks CRP as a precise objective index of inflammatory activity and surrogate of underlying cytokine stimulus and a sensitive marker of underlying systemic inflammation [35]. It has been demonstrated that coagulation indices including fibrinogen, von Willebrand factor, and plasminogen activator activity are increased in BD and Hcy is thought to promote the clotting cascade via several actions including inactivation of protein C, activation of coagulation factor V, increased vascular smooth muscle cell proliferation, and inhibition of thrombomodulin [15]. CRP is thought to have effects into the mechanism linking inflammation and coagulation [35]. Hcy is thought to induce proinflammatory cytokines [24, 36–38]. In vasculitis such as BD, endothelial-leukocyte interaction is an important part of the inflammation [21] and Hcy has been reported to increase endothelial-leukocyte interaction [13]. Hcy has been shown in vivo and in vitro to promote inflammatory process such as the adhesion of neutrophils to endothelial cells as well as the release of the inflammatory cytokine IL-8 and monocyte chemoattractant protein-1 (MPC-1) [39]. Hcy was shown to enhance the cytokine-stimulated expression of endothelial cell adhesion molecules and monocyte and T-cell adhesion to endothelial cells [39]. Adhesion molecules are characterized not only by their ability to cause directed migration of leukocytes into inflamed tissue, but they may also have biological effects on other cell types, such as endothelial cells, fibroblasts, and smooth muscle cells [40]. Hcy was shown to promote TNF-*α* mediated induction of vascular cell adhesion molecule-1 (VCAM-1) in endothelial cells [41]. With respect to innate inflammatory markers such as TNF-*α* and CRP, serum Hcy has been shown to contribute to innate immune response and to be a determinant of TNF-*α* in hypertensive patients [36]. In addition, Hcy has been shown to enhance inflammation markers in murine models [42]. The correlation of Hcy levels with nitric oxide has been shown in patients with BD [15]. Hcy is known to increase nitric oxide production in vascular smooth muscle cells and nitric oxide is known to be released as a response to systemic immunoinflammatory diseases [15]. A study demonstrated that targeting the TNF pathway significantly decreased Hcy and CRP levels in patients with psoriatic arthritis and in this study, CRP showed a positive correlation with Hcy at baseline [43]. CRP has been shown to correlate with Hcy in BD [19]. Hcy has been suggested to be related to the markers of inflammation and its elevated levels as a result of immunosuppressive treatment [19]. Indeed, immunosuppressive treatments may affect serum levels of Hcy and TNF-*α* [44]. This is the reason why we studied with the patients who were not taking any medication regarding BD. This definite selection decreased the number of our study population as a study limitation. In our study, serum levels of active BD patients with vascular involvement, oral ulcer, arthritis/arthralgia, EN/PPE and the presence of positive pathergy test were found significantly increased compared to inactive BD patients with the same clinical symptoms. Therefore, serum levels of Hcy were found to explain the wide clinical spectrum of the activity of the specific individual symptoms.

In summary, Hcy and CRP have effects into the mechanism linking inflammation and coagulation and Hcy is thought to induce proinflammatory cytokines including TNF-*α*, which is known to increase chemotactic and phagocytic activities of neutrophils, endothelial-leukocyte interaction, and CRP levels. We found a high correlation between serum levels of Hcy and TNF-*α* and Hcy was found to explain the wide clinical spectrum of the activity of the disease and specific individual symptoms. Taking all these findings into account, a theory might be put forward which is open to further in vivo and in vitro studies; Hcy-induced inflammation may be responsible for tissue damage as well as endothelial dysfunction in BD and enhanced Hcy may account for the differences observed between clinically distinct subgroups. Lowering Hcyby selected vitamin supplements or TNF blockade may be the future direction of prevention from developing the risk of thrombosis and vasculitis of different organ systems and thereby activation of BD disease.

## 5. CONCLUSION

Our data identified Hcy as a risk factor for the development of not only vascular and ocular involvement but also most clinical features that are known to be associated with vasculitis in BD. In addition, serum Hcy showed a strong association of disease activity and correlated with BDAI scores and the number of active clinical manifestations increased. Therefore, Hcy might be assumed as a reliable marker for the activity of disease. Hcy was found to be the best predictor of TNF-*α* and positively correlated with inflammatory markers. Our findings suggest that Hcy might contribute to the pathogenesis of BD by inducing inflammation. Further investigation in clinical and experimental studies is required to conclusively demonstrate the association between Hcy and inflammatory pathways.

## Figures and Tables

**Table 1:** Comparision of serum homocysteine, TNF-*α*, CRP, and ESR values and BDAI scores between groups.

		Homocysteine (*μ*mol/L)	TNF-*α* (pg/mL)	ESR (mm/h)	CRP (mg/L)	BDAI scores
		Median (range)				

Group I (active BD) (*n* = 34)		20.95 (13.7–39.25)	93.5 (30–228)	24 (2–95)	20.5 (3–400)	16 (9–25)
Group II (inactive BD) (*n* = 36)		13 (6.43–18)	19 (15–30)	27 (8–49)	6.65 (3–34)	2 (1–5)
Group III (healthy control) (*n* = 33)		8 (5–8)	0 (0–18)	6 (2–18)	3.1 (3–10)	

Significance for groups	I-II-III	^a^ *P* = .00*	^a^ *P* = .00*	^a^ *P* = .00*	^a^ *P* = .00*	^c^ *P* = .00*
	I-II	^b^ *P* = .00*	^b^ *P* = .00*	^b^ *P* = .001*	^b^ *P* = .00*	
	I-III	^b^ *P* = .00*	^b^ *P* = .00*	^b^ *P* = .00*	^b^ *P* = .00*	
	II-III	^b^ *P* = .00*	^b^ *P* = .00*	^b^ *P* = .008*	^b^ *P* = .00*	

^a^ P* Significantly different by Kruskal-Wallis test (*P* < .05). ^b^ P* Significantly different by Bonferroni corrected Mann-Withney *U* test (*P* < .0167). ^c^ P* Significantly different by Mann-Withney *U* test (*P* < .05). Data are presented as median values and their individual ranges within parentheses.

**Table 2:** The relationship between variables.

	Hcy	ESR	CRP	TNF	Total†	BDAI
Spearman correlation coefficient and *P*-values						

Hcy		*r* = 0.561	*r* = 0.645	*r* = 0.889	*r* = 0.694	*r* = 0.836
		*P* = .00	*P* = .00	*P* = .00	*P* = .00	*P* = .00

ESR	*r* = 0.561		*r* = 0.421	*r* = 0.538	*r* = 0.304	*r* = 0.408
	*P* = .00		*P* = .00	*P* = .00	*P* = .011	*P* = .00

CRP	*r* = 0.645	*r* = 0.421		*r* = 0.670	*r* = 0.549	*r* = 0.583
	*P* = .00	*P* = .00		*P* = .00	*P* = .00	*P* = .00

TNF	*r* = 0.889	*r* = 0.538	*r* = 0.670		*r* = 0.842	*r* = 0.924
	*P* = .00	*P* = .00	*P* = .00		*P* = .00	*P* = .00

Total†	*r* = 0.694	*r* = 0.304	*r* = 0.549	*r* = 0.842		*r* = 0.842
	*P* = .00	*P* = .011	*P* = .00	*P* = .00		*P* = .00

BDAI	*r* = 0.836	*r* = 0.408	*r* = 0.583	*r* = 0.924	*r* = 0.842	
	*P* = .00	*P* = .00	*P* = .00	*P* = .00	*P* = .00	

Total† number of active clinical manifestations for each patient.

**Table 3:** The significance of the association of Hcy with versus without individual clinical manifestations and pathergy reaction.

		Hcy levels (*μ*mol/L) in patients with		Hcy levels (*μ*mol/L) in patients without		
Manifestations	No. of Patients	Median	(Range)	Median	(Range)	*P*-values
Oral ulcer	31	18.03	(8–39.3)	14.28	(6.4–29)	*P* = .003*
Genital ulcer	23	18.1	(11.9–38.1)	14.28	(6.4–39.3)	*P* = .005*
Ocular lesion	18	21.59	(14–39.3)	14.15	(6.4–38)	*P* = .00*
Vascular lesion	37	19	(11.9–39.3)	13.75	(6.4–26)	*P* = .00*
Arthritis/arthralgia	25	16	(6.4–39.3)	15	(8–38.1)	*P* = .99
EN/PPE	31	16	(8.1–38.1)	14.88	(6.4–39.3)	*P* = .306
(+) pathergy	35	17.2	(7.9–38.1)	14	(6.4–39.3)	*P* = .007*

P* < .05 by Mann-Whitney *U* test (statistically significant with versus without clinical manifestations). Data are presented as median values and their individual ranges within parentheses.

**Table 4:** The significance of the association of TNF-*α* with versus without individual clinical manifestations and pathergy reaction.

		TNF-*α* levels (pg/mL) in patients with		TNF-*α* levels (pg/mL) in patients without		
Manifestations	No. of patients	Median	(Range)	Median	(Range)	*P*-values
Oral ulcer	31	70	(15–228)	20	(15–150)	*P* = .00*
Genital ulcer	23	54	(17–228)	21	(15–228)	*P* = .00*
Ocular lesion	18	99	(30–228)	21	(15–180)	*P* = .00*
Vascular lesion	37	84	(15–228)	21	(15–145)	*P* = .00*
Arthritis/arthralgia	25	38	(15–228)	28	(15–228)	*P* = .49
EN/PPE	31	45	(15–228)	22	(15–228)	*P* = .086
(+) pathergy	35	45	(15–228)	21	(15–228)	*P* = .00*

P* < .05 by Mann-Whitney *U* test (statistically significant with versus without clinical manifestations). Data are presented as median values and their individual ranges within parentheses.

**Table 5:** The significance of the association of CRP with versus without individual clinical manifestations and pathergy reaction.

		CRP levels (mg/L) in patients with		CRP levels (mg/L) in patients without		
Manifestations	No. of patients	Median	(Range)	Median	(Range)	*P*-values
Oral ulcer	31	12.4	(3–400)	8.7	(3–43)	*P* = .159
Genital ulcer	23	18	(3–100)	8.4	(3–400)	*P* = .001*
Ocular lesion	18	19.25	(3–400)	8.5	(3–68)	*P* = .001*
Vascular lesion	37	20.5	(3–400)	8.4	(3–51)	*P* = .001*
Arthritis/arthralgia	25	8.4	(3–400)	12.9	(3–400)	*P* = .201
EN/PPE	31	12.9	(3–400)	8.9	(3–400)	*P* = .043*
(+) pathergy	35	14.8	(3–400)	8.7	(3–400)	*P* = .016*

P* < .05 by Mann-Whitney *U* test (statistically significant with versus without clinical manifestations). Data are presented as median values and their individual ranges within parentheses.

**Table 6:** The significance of the association of ESR with versus without individual clinical manifestations and pathergy reaction.

		ESR levels (mm/h) in patients with		ESR levels (mm/h) in patients without		
Manifestations	No. of patients	Median	(Range)	Median	(Range)	*P*-values
Oral ulcer	31	21	(2–95)	12	(2–70)	*P* = .113
Genital ulcer	23	22	(2–95)	14	(2–60)	*P* = .121
Ocular lesion	18	22	(2–86)	14	(2–95)	*P* = .291
Vascular lesion	37	20	(2–70)	14	(2–95)	*P* = .642
Arthritis/arthralgia	25	22	(3–60)	14	(2–95)	*P* = .317
EN/PPE	31	24	(2–95)	12	(2–70)	*P* = .070
(+) pathergy	35	22	(2–95)	14	(3–49)	*P* = .173

P* < .05 by Mann-Whitney *U* test (statistically significant with versus without clinical manifestations). Data are presented as median values and their individual ranges within parentheses.

**Table 7:** Predictors of plasma TNF-*α* level: regression analysis.

Predictor of TNF-*α*	*R* ^2^	Beta	*P*-value
Hcy	0.88	6.45	*P* = .00
CRP	0.39	0.559	*P* = .00
ESR	0.146	1.15	*P* = .00

Dependent variable: TNF-*α*.

**Table 8:** Comparison of Hcy intragroups of active and inactive patients according to presence of vascular involvement.

		Hcy levels (*μ*mol/L)		
		Median	Range	*P-* values
Active BD (*n* = 34)	With vascular involvement (*n* = 20)	26	19–39.25	.00*
	Without vascular involvement (*n* = 14)	15.98	13.7–26	

Inactive BD (*n* = 36)	With vascular involvement (*n* = 17)	14.28	11.94–18	.00*
	Without vascular involvement (*n* = 19)	10.75	6.43–16	

P* < .05 by Mann-Whitney *U* test. Data are presented as median values and their individual ranges.

**Table 9:** Comparison of Hcy between groups of active and inactive patients according to the presence of individual clinical manifestations and positive pathergy.

	No. of patients		Hcy levels (*μ*mol/L)		*P*-values
	Active	Inactive	Active	Inactive	
Vascular involvement	20	17	26 (19–39.25)	14.28 (11.94–18)	.00*
Oral ulcer	26	5	20.85 (13.7–39.25)	11.94 (8–14)	.00*
Arthritis/arthralgia	14	11	19 (13.7–19)	10.83 (6.43–17)	.00*
EN/PPE	21	10	19 (13.7–38.1)	12.36 (8.1–16)	.00*
Positive pathergy	24	11	20.85 (13.7–38.1)	14.28 (7.91–17.21)	.00*

P* < .05 by Mann-Whitney *U* test. Data are presented as median values and their individual ranges.
